# The pioneers of Vietnam’s epidemiological transition: an ethnographic study of pregnant women’s experiences of gestational diabetes

**DOI:** 10.1080/16549716.2024.2341521

**Published:** 2024-05-02

**Authors:** Tine M. Gammeltoft, Thi Ai Nguyen, Thi Kim Dung, Ngoc-Anh Thi Dang, Thi Minh Phuong Nguyen, Van Tien Nguyen, Ib C. Bygbjerg

**Affiliations:** a Department of Anthropology, University of Copenhagen, København, Denmark; b Department of Health Management & Organization, Faculty of Public Health, Thai Binh University of Medicine and Pharmacy, Hue, Vietnam; c Department of Epidemiology, Faculty of Public Health, Thai Binh University of Medicine and Pharmacy, Hue, Vietnam; d Department of Environmental Health, Faculty of Public Health, Thai Binh University of Medicine and Pharmacy, Hue, Vietnam; e Department of Health Sociology, Faculty of Public Health, Thai Binh University of Medicine and Pharmacy, Hue, Vietnam; f Department of Public Health, Global Health Section, University of Copenhagen, København, Denmark

**Keywords:** Digital health, family environment, health services, phenomenological anthropology, pregnancy, self-care

## Abstract

**Background:**

Gestational diabetes mellitus (GDM) is an abnormal glucose metabolism diagnosed during pregnancy that can have serious adverse consequences for mother and child. GDM is an exceptional health condition, as its management serves not only as treatment but also as prevention, reducing the risk of future diabetes in mother and child.

**Objectives:**

This qualitative study aimed to explore how pregnant women experience and respond to GDM, focusing particularly on the role of the family environment in shaping women’s experiences.

**Methods:**

The research was carried out in Vietnam’s Thái Bình province in April–May 2023. We conducted in-depth ethnographic interviews with 21 women with GDM, visiting them in their homes. Our theoretical starting point was phenomenological anthropology, and the data were analysed using a thematic analysis approach.

**Results:**

At the centre of women’s experiences was the contrast between GDM as a biomedical and a social condition. Whereas GDM was biomedically diagnosed and managed in the healthcare system, it was often deemed insignificant or non-existent by family members. This made GDM a *biomedically present* but *socially absent* health condition. This paradox posed challenges to women’s GDM self-care, placing them in pioneering social positions.

**Conclusions:**

The biomedical presence yet social absence of GDM turned women into pioneers at biomedical, digital, epidemiological, and family frontiers. This article calls for appreciation of pregnant women’s pioneering roles and for health systems action to involve women and families in the development of GDM policies and programmes at a time of sweeping global health changes.

## PAPER CONTEXT

**Main findings:** Vietnamese women’s experiences of gestational diabetes were affected by social splits between clinic and home; between biomedical and family worlds.

**Added knowledge:** Gestational diabetes places pregnant women in Northern Vietnam in pioneering roles on biomedical, digital, epidemiological, and family frontiers.

**Global health impact for policy and action:** Pregnant women should be involved in the development of policies and programmes addressing gestational diabetes, with particular attention to the connections between clinical and family worlds.

## Background

Gestational diabetes mellitus (GDM) – an abnormal glucose metabolism diagnosed during pregnancy – is a growing public health problem across the world [1]. Women with GDM have elevated risks of pregnancy complications and of giving birth to large for gestational-age infants, and both mother and child are at increased risk of developing type II diabetes later in life [2,3]. This turns GDM into an exceptional health condition, as its management serves not only as treatment but also as prevention, reducing the risk of future diabetes in both mother and child. Pregnancy and postpartum periods are thus important windows of opportunity for the prevention of diabetes in two generations – yet making use of this opportunity for pre-primary prevention demands that GDM is detected and appropriately managed [4,5]. In this context, previous studies have pointed to the critical importance of women’s GDM self-care practices [6,7], while also emphasising the challenges to self-care that often arise in resource-constrained settings, such as lack of adequate professional or informal support [8–10].

This study was conducted in Vietnam, where the prevalence of all forms of diabetes is currently rapidly rising [11,12]. Previous studies have found a GDM prevalence rate in Vietnam of 22.8%, close to the estimated prevalence for Southeast Asia [12,13]. GDM is recognised by Vietnam’s Ministry of Health as a significant public health problem, and national guidelines on prevention and management are in place, forming part of the country’s efforts to prevent and control non-communicable diseases (NCDs) [14]. Despite these policy intentions, systematic screening for GDM is not undertaken, and counselling services are not well established. Further, little is known about how health providers, pregnant women, and families perceive and handle the condition. This ethnographic study, therefore, set out to investigate how pregnant women and their family members experience and respond to GDM, focusing particularly on the implications for self-care.

In approaching women’s experiences as matters of pioneering, we are inspired by the ground-breaking work by anthropologist Rayna Rapp who framed pregnant women’s use of new reproductive technologies as moral pioneering [15]. We expand on Rapp’s work by attending not only to the social and clinical but also the digital and epidemiological dimensions of pregnant women’s pioneering roles in a time of radical global health changes. In terms of theory, our starting point is phenomenological anthropology, an approach that seeks to understand people’s lives as they experience them themselves [16,17].

## Methods

### Study setting

The research was conducted in northern Vietnam’s Thái Bình province in April–May 2023. Located in the Red River delta, Thái Bình’s population includes around two million people who live mainly from wet-rice cultivation and industrial work. In Thái Bình as elsewhere in Vietnam, diabetes is a growing public health problem – a decade ago seen as ‘a disease of the rich’ but now prevalent across population groups. Previous research in this province has found a GDM prevalence of 20.2% [18], while preliminary findings from our research show that 28.2% of pregnant women have GDM, assessed according to WHO criteria [19].

The prevalence of GDM in Thái Bình must be seen in the context of rapidly changing socio-economic conditions: over a few decades, Vietnam has leaped out of poverty and global isolation, reducing poverty rates from almost 60% in 1993 [20] to 4.4% in 2021 [21]. These large-scale developments have fundamentally changed daily lives, altering eating habits; transportation/movement patterns; and increasing exposure to air pollution [22]. GDM is, in this perspective, a somatic marker of rapidly changing social conditions: women with GDM can be seen as *epidemiological pioneers* whose bodies register large-scale social transformations.

In Thái Bình, screening for GDM has been offered by private clinics since 2018 and by provincial-level hospitals since 2019. At the time of our research, women had to pay out of pocket for GDM screening, and the service was not universally used. As maternity care staff in the province lacked expertise in endocrinological conditions, women with GDM were referred to the provincial general hospital for re-testing and counselling by endocrinologists. After the initial diagnosis, follow-up GDM management was not routinely offered within the antenatal care system.

### Study design and sample

This qualitative study forms part of an ongoing, interdisciplinary intervention research project investigating GDM self-care among pregnant women [23]. The project is a Vietnamese-Danish research partnership with a strong emphasis on research capacity building. The ethnographic component includes an extended case study involving 21 women with GDM who were followed over time, from the time of diagnosis until 12 months postpartum.

The 21 women were recruited from the project’s larger sample of 233 women diagnosed with GDM through an oral glucose tolerance test [19]. In April 2023, telephone calls were made to all women who by that time had been diagnosed with GDM. Of 105 women contacted, 21 agreed to take part in the ethnographic study. When invited to take part, many women refused either due to full-time work and lack of time or due to doubts about the research team’s intentions, as pregnant women are often the targets of commercial agents seeking to sell pregnancy merchandise or formula milk.

All 21 women were married, with 9 living in two-generational, 10 in three-generational, and 2 in four-generational households. In four cases, the husband was working in another province and only at home during weekends and/or holidays. Nine women were pregnant with their first child, three with their second, seven with their third, one with her fourth, and one with her fifth child. One had GDM in a previous pregnancy, and two suspected they might have had GDM before. The women were aged 20–47, with an average of 32.3 years. They worked in different occupations, including factory workers, teachers, office workers, and health workers. Table 1 provides an overview of the socio-economic characteristics of study participants, alongside the characteristics of the 233 women with GDM in the full sample.

**Table 1.** Descriptive characteristics of participants in the ethnographic study and the full study.

Characteristics		Participants in ethnographic study (*N* = 21)	% of total	Participants in full study (*N* = 233)	% of total
Age	Mean (range)	32.3 (20–47)		30.5 (–1947)	
Education	Primary school	0	0	1	0.4
	Secondary school	2	9.5	27	11.6
	High school	8	38.1	55	23.6
	Intermediate/college/university/postgraduate	11	52.4	150	64.4
Occupation	Factory worker	7	33.3	67	28.8
	Teacher/office worker/health staff	9	42.9	115	49.3
	Self-employed	5	23.8	21	9.0
	Student/not working	0	0	30	12.9
Family economic status (self-assessed)	Poor/near poor	0	0	3	1.3
	Medium	20	95.2	226	97.0
	Wealthy	1	4.8	4	1.7
Marital status	Married	21	100	230	98.7
	Unmarried/divorced	0	0	3	1.3
Household	Two-generational	9	42.9	106	45.5
	Three-generational	10	47.6	123	52.8
	Four-generational	2	9.5	4	1.7
Number of living children	0	9	42.8	82	35.2
	1	3	14.3	84	36.1
	2	7	33.3	55	23.6
	3	1	4.8	10	4.3
	4	1	4.8	2	0.9

Overall, as regards age, education, economic status, and household composition, the 21 women taking part in the ethnographic study did not differ substantially from the larger group of 233 women. The main differences between the two groups were that more women in our ethnographic sample were pregnant with their first child and more were self-employed. These differences between the two groups are unsurprising: women without children and women working from home are likely to find it easier to find time for an interview than mothers of small children with work outside the home. We discuss the possible implications for the analysis of these minor differences in the discussion section below.

### Data collection: the extended case study

A few weeks after their diagnosis, we conducted in-depth ethnographic interviews with the women in their homes. Each home visit lasted for around 2 hours and was carried out by the first author and one or two co-authors. Depending on the women’s preferences, participants were either only the woman herself (12 cases) or the woman and other family members such as husband, parents, or parents-in-law (nine cases). The interviews were based on an open-ended interview guideline which addressed the main research topics while also allowing ample room for improvisation. Women and their family members seemed eager to talk and share their experiences, and it was our impression that the research team was met with considerable trust and openness. The medical university hosting the research project is well known throughout the province and considered a prestigious and trustworthy institution, and this status seemed to facilitate contact with our participants. Throughout the research, detailed ethnographic field notes were written, including observations of bodily expressions and social interactions [24]. The conversations were complemented by photographs taken during the home visit. All interviews were voice recorded, transcribed, and systematically coded using a thematic coding list. The data were stored on an online platform.

## Results

Our findings show that Vietnamese women’s GDM experiences turned them into pioneers at epidemiological, social, biomedical, and digital frontiers. In the following, we highlight the processes through which women come to be placed in pioneering roles, starting with a case example:

### Case illustration

*Thảo’s case: ‘Oh, Heavens, GDM* *– does anyone not have that during pregnancy?’*

In April 2023, Thảo was 40 years old and 29 weeks pregnant with her third child. She lived in a small town about an hour’s drive from the provincial city. When we arrived, Thảo’s two daughters, aged 5 and 16, were still at school, and the house was quiet. ‘I test my blood sugar one hour before breakfast and two hours after breakfast,’ she told us. ‘I work at a company. In the beginning, I tested at noon too, but I felt uncomfortable about it, so now I just test morning and evening. There are days when it’s above eight dots. I feel bewildered.’

Since a GDM test taken at a private clinic a few weeks earlier indicated high levels of glucose in her blood, Thảo had been feeling uncertain, anxious, and confused. Doctors at the clinic encouraged her to seek expert help at the city’s general hospital, but as she did not have anyone to drive her, she did not go. Instead, she searched for information on the Internet. This search had been a lonely endeavour: working in the army, Thảo’s husband was away from their home most of the time, and she had been studying the Internet information on her own. When we asked her what kind of information she found, Thảo took her iPhone from the table and showed us a Messenger thread. Here, she had copied results from her Google search into a conversation with her husband: ‘GDM can increase the risk of miscarriage, stillbirth, preterm birth, high blood pressure, (…) and C-section’, the text said. To Thảo, this was frightening. ‘But my husband did not care,’ she said. ‘He told me to just relax and eat.’ When talking to other family members, such as her mother and sisters, she met similar reactions. ‘Oh, Heavens, GDM – does anyone *not* have that during pregnancy,’ her younger sister exclaimed when she heard about Thảo’s positive test. ‘There is no need to pay attention to that (*không phải quan tâm đâu*).’ Emphasising the harmlessness of the condition, her family members encouraged her to ignore the GDM diagnosis and continue living as before.

Thảo described her relatives’ limited attention to her diagnosis with an expression of deep frustration. She felt intensely worried, yet people around her did not seem to share her feelings. This placed her uneasily between biomedical messages emphasising the dangers of GDM and advice from family members stressing the condition’s harmlessness.

Not all women in our sample experienced as stark a contrast between biomedical and family worlds as Thảo did: in some cases, the woman herself would, like her family members, feel relatively untroubled by the GDM diagnosis, while in other cases, family members shared the woman’s worries. What the women had in common was the experience of GDM being articulated in very different ways in biomedical and family worlds.

### GDM in biomedical worlds: a threat to mother and child

When talking about their GDM diagnosis, many women described feelings of disorientation and doubt. Most had received no or very limited counselling when the GDM diagnosis was made: in most cases, antenatal care staff would simply state in brief terms that elevated glucose levels had been found, saying that GDM is a dangerous condition and that it was important to reduce intake of sugar and white rice. When encouraged to seek specialist care at the provincial general hospital, some called the endocrinologist or went to the hospital for re-testing and counselling. Most women, however, opted not to seek such care due to transportation difficulties, financial costs, or feelings of inferiority. Confronted with medical hierarchies, they felt, as many put it, embarrassed (’*ngại’*) to take the initiative in seeking care [25,26]. In Thanh’s words: ‘I wish I would not have to go and ask by myself. I wish the doctors would offer me information. I feel so embarrassed to have to ask myself. I wish I could have received counseling right there, at the antenatal care clinic.’

Like Thảo and Thanh, many therefore returned home with numerous unanswered questions, feeling uncertain about how to understand and act on their diagnosis. Thirty-two-year-old Thơm expressed this in words that resonated with the sentiments expressed by many other women: ‘I worry about how to control my diet and how to control my blood sugar so it’s stable. I’m also scared of complications, especially since the fetus may be very large. I’m also worried because few people around me have had this, so I have very little information.’ Like other women, Thơm expressed a wish for more detailed GDM counselling: ‘Having to ask for counseling myself makes me feel the doctor does not care about me. The doctors told me to adjust my diet, but I don’t know how to do this. I never eat sweet things, so I don’t know what else I can do?’

As a consequence of this limited support, the Internet often became a primary source of information [27]. Searching via Google, the women studied official websites such as that of Vietnam’s Ministry of Health while also reading articles that appeared during Internet searches. Some joined Facebook groups where members could ask questions regarding GDM and other pregnancy-related conditions, some followed doctors on TikTok who explained about the disease while also advertising for commercial products, and some downloaded pregnancy apps where information was offered. Many found the information from the Internet confusing and frightening. Thu, for instance, said, ‘I learnt that GDM can lead to many complications. The child can become very fat, and perhaps there will be problems with its brain. I’m scared that my child will be like that. I don’t know what to do anymore.’ Some women were able to discuss the Internet information with their husbands or other family members – but many digested it alone. In An’s words, ‘I did not tell my parents anything. This is something I know about myself. I read on the Internet, and I learned that I should reduce carbohydrates and sweets, and that I should eat more cereals … To be honest, I did not receive any counselling from anyone.’ As many women felt uncertain about the trustworthiness of Internet sources, Internet information often seemed to deepen their feelings of disorientation and doubt. In Vũ’s words: ‘I sometimes search on the Internet. But I find that they write so many random things. No one is checking what they write, so I don’t trust it. On YouTube, so much nonsense is posted.’

Biomedical information came, in short, in many forms – sometimes via face-to-face clinical encounters with physicians and sometimes via the Internet. Regardless of source, a key biomedical message was that GDM is a dangerous condition, potentially very harmful to mother and child. Hearing this, the women reacted in different ways: some felt anxious and nervous, as illustrated by Thảo’s case, while others opted not to take biomedical messages too seriously, trusting that they and their child-to-be would be fine. Women who opted to define GDM as a benign condition often found support in reassuring stances from family members.

## GDM in the family: an insignificant condition

‘When my husband came home, he said, “Nowadays, does anyone *not* have GDM?” He encouraged me to simply eat normally, to take it easy and not worry too much. This does not matter, he says. He encourages me’ (Trung, 29 years old). Like Trung, when receiving the GDM diagnosis, most women immediately shared the news with their husbands [28]. Most husbands responded by stressing the ordinariness of the condition, telling their wives not to worry about it. The term most commonly used in husbands’ responses was *kệ*, which can be translated as ‘just ignore it.’ Some women took the term *kệ* as a gesture of care, feeling that their husbands tried to support them by encouraging them not to worry, while others felt that their husband was abandoning them at a difficult time, expecting them to take sole responsibility for the pregnancy. Women who felt supported often described how their husbands joined them in GDM self-care, for instance by going for walks together in the evening or altering daily diets to eat more brown rice and vegetables. Women who felt abandoned often felt that their husbands lacked interest in the pregnancy, seeing childbearing as a ‘women’s issue.’ Học, instance, said, ‘He does not care. Whenever I try to talk to him, he just embraces his phone … In the evening, we sit each with our phones. I listen to some music, and then I go to bed.’

When informed about the woman’s GDM diagnosis, family members would often refer to other people’s experiences with GDM, recalling cases of women who had been diagnosed with GDM without any apparent implications for the mother or child, and encouraging the woman not to think too much about the diagnosis. As 31-year-old Hạnh said, ‘My husband asked his friend. His friend’s wife also had GDM, and at birth the child was normal. So, my husband tells me to ignore this and just eat, there is nothing to be afraid of.’ In some cases, women opted to trust this advice, taking comfort in their relatives’ words, while in others, it seemed to only deepen their feelings of worry and isolation.

Many of the women lived in extended families, sharing a household with their own or their husband’s parents. Women living in extended families usually told their elders about the GDM diagnosis, while women living in nuclear families were more inclined to keep the diagnosis to themselves, feeling that their elders lacked insight into this new health problem, and also not wanting to burden them with uncertainties regarding the pregnancy. In extended families, the grandparents-to-be were in most cases intensely involved in pregnancy care, striving to care for their daughter or daughter-in-law by releasing her from everyday household tasks and supporting her to eat appropriately and get enough rest. Twenty-four-year-old Lanh, for instance, said: ‘My mother-in-law is looking so much forward to having a grandchild, so she allows me to rest all the time. I do not have to do anything to help, my mother-in-law does everything for me. All I have to do is turn on the rice cooker and prepare some soup before my mother-in-law comes home from work. I do not have to do any heavy work at all.’ Yet such parental pregnancy care did not always include support to handle GDM. When parents/parents-in-law heard about the woman’s GDM, they would most often declare like husbands that GDM is a transient and harmless condition that disappears after birth. In Vũ’s words: ‘They tell me I should “just eat” (*cứ ăn đi*). But how can I just eat? I have to make sure I’m stable, and I must also make sure that the weight of the little one is OK.’

In many cases, these contradictory stances – health systems messages warning them of the risks of GDM, family members dismissing its importance – made women feel lonely and abandoned. Thơm, for instance, said, ‘in my home, no one understands this. I’m the one who is pregnant, so I’m the one who feels most worried. Everyone else is outside (*mọi người chỉ là cái bên ngoài thôi*), it’s as if they don’t understand and cannot share this with me.’ Other women, in contrast, felt supported by their family members, taking family members’ dismissal of GDM as an expression of care, concern, and support.

## Discussion

Most studies of GDM self-care focus on the individual woman’s perceptions and practices, with less attention paid to the family/household environment [6,29]. Yet our findings indicate that relations between clinical and domestic settings are of critical importance, shaping pregnant women’s orientations and capacities for self-care. For many women in this study, the biomedical presence yet domestic absence of GDM created tension, turning self-care into a stressful endeavour, and placing the women in lonely and pioneering positions at biomedical, digital, and social frontiers.

Firstly, the women were *biomedical pioneers*, struggling to determine how to interpret and act on a new biomedical diagnosis; one that made many feel disoriented and confused. Previous studies have documented similar problems, pointing to widespread experiences of anxiety, fear, and disorientation when a GDM diagnosis is made, and stressing the importance of professional support at this time [29–31]. For women in our study, the disorientation was corroborated by limited health system support: rather than being enrolled in follow-up schemes to help them through the pregnancy, the women were largely left to themselves, having to find their ways within an antenatal care system without established procedures for GDM management and with highly limited access to counselling. This turned the women into pioneers within new and uncertain biomedical terrains in ways that are comparable to the pioneering that took place in the early days of the global HIV/AIDS epidemic [32].

Secondly, the women were *digital pioneers*, making intensive use of online information to compensate for the limited health system support. Other studies have documented similar tendencies for online information to take the place of professional support when healthcare services are lacking or limited [10]. Self-retrieved online information in a situation of limited professional/clinical counselling may, however, deepen women’s feelings of disorientation, due to conflicting information and uncertainties regarding whether online information can be trusted. The scepticism regarding online information expressed by women in our study resonates with research pointing to suboptimal quality of online health information and warning against laypeople’s use of such information [33].

Thirdly, the women in this study were *social pioneers*. Being among the first in their families and social communities to receive a GDM diagnosis, they broke new ground, searching for ways to integrate an asymptomatic and elusive medical condition into daily lives. As family members tended to stress the transience and insignificance of GDM, this often proved challenging. GDM was a *disease* – a biomedically diagnosed condition – yet it was not always an *illness*, understood as a subjectively felt problem [34]. Further, family responses to this elusive condition must be seen in the context of culture and history. In Vietnam, traditional pregnancy advice – rooted in times of scarcity and hunger – urges pregnant women to stay mentally balanced and worry-free while also encouraging them to rest much and eat plenty to ensure foetal growth [35,36]. Such long-held ideals for good pregnancy care tend to conflict with biomedical GDM advice. Similar clashes between long-established cultural practices and new biomedical GDM lifestyle requirements have been described in studies from other countries undergoing rapid social change [37,38]. In social worlds too, then, women with GDM found themselves at the frontlines, stepping into new and unknown terrains. They longed for accompaniment – socially and clinically – but did not always find it.

### Study strengths and limitations

The study ensured validity through its collaborative methodological approach which was characterised by a high degree of researcher reflexivity [39]. Reflexivity was ensured through joint creation of research questions; careful notetaking, including observations of social interactions, environments, and atmospheres as well as reflections on researcher–participant interactions; and continuous discussions among the researchers on the team of the meaning and importance of findings. Validity was also enhanced by spending considerable time in the women’s homes – a place they felt at ease during the pregnancy – and by working with an open-ended and flexible research agenda that allowed ample space for respondents’ questions and concerns.

As mentioned above, only 21 of 105 women contacted accepted the invitation to take part in the ethnographic study, and there were slightly more first-time mothers and self-employed women in this ethnographic sample as compared to the overall sample of 233 women with GDM. Yet since our main finding – the split between biomedical and social worlds – was pertinent and manifest regardless of parity and occupation, we do not find it likely that the sampling process has introduced selection bias with consequence for the analysis.

Despite its strengths, the study also had limitations: the research was conducted in one rural province of Vietnam, and our findings do not capture the experiences of women with GDM living in other parts of the country such as highly urbanised or mountainous regions. As GDM is a relatively novel health problem in Vietnam, we recommend that similar research be carried out in other regions of the country to explore the generalisability of the results.

## Conclusions

Although GDM is a transitory condition, its sequels do not always disappear. The critical importance of GDM self-care for maternal and child health and prevention of future diabetes is well established, yet such self-care often meets challenges. This study has documented how a split between biomedical and family responses to GDM formed the ground for Vietnamese women’s experiences and self-care practices: in biomedical worlds, GDM was represented as a serious threat to mother and child, whereas in family worlds, it was often characterised as an insignificant and transient condition. In many cases, this split turned GDM into a lonely experience, placing pregnant women at epidemiological, biomedical, digital, and social frontiers, and rendering self-care difficult.

This article calls for further attention in research and healthcare programming to the relations between clinical and family worlds and for health policy appreciation of pregnant women’s pioneering roles in the GDM field. There is an urgent need for further, in-depth qualitative research on the perspectives of husbands and other family members involved in informal GDM care, in Vietnam as well as other low- and middle-income countries, as well as for qualitative studies of the viewpoints and practices of GDM healthcare providers. Further, systematic quantitative research on the proportion of women with GDM diagnosed and offered appropriate counselling and follow-up would contribute significantly to the knowledge base needed to enhance maternity care in this field.

In settings such as the Vietnamese where GDM is a novel reproductive health problem, the need to include women’s perspectives and experiences in the development of policies and guidelines for antenatal care achieves further urgency. Health systems strengthening in this field should include health provider training; systematic public health information on GDM prevention, targeting not only pregnant women but the public at large; and free and easy access to GDM screening, including supportive counselling and follow-up for those diagnosed with GDM. Combining systematic health systems strengthening with clear public health information in the GDM field would contribute to aligning social worlds of families with clinical worlds of biomedicine, thereby enhancing pregnant women’s self-care capacities and supporting their self-care practices.
